# Phase 1 trial of ADI‐PEG 20 and liposomal doxorubicin in patients with metastatic solid tumors

**DOI:** 10.1002/cam4.4446

**Published:** 2021-11-28

**Authors:** Shuyang Yao, Filip Janku, Kimberly Koenig, Apostolia Maria Tsimberidou, Sarina Anne Piha‐Paul, Nai Shi, John Stewart, Amanda Johnston, John Bomalaski, Funda Meric‐Bernstam, Siqing Fu

**Affiliations:** ^1^ Department of Investigational Cancer Therapeutics Houston Texas USA; ^2^ Department of Thoracic Surgery Xuanwu Hospital Capital Medical University Beijing China; ^3^ Department of Breast Medical Oncology Houston Texas USA; ^4^ Department of Pathology The University of Texas MD Anderson Cancer Center Houston Texas USA; ^5^ Polaris Pharmaceuticals, Inc. San Diego California USA

**Keywords:** ADI‐PEG 20, advanced solid tumor, arginine deprivation, argininosuccinate synthetase, breast carcinoma, liposomal doxorubicin

## Abstract

**Background:**

Arginine depletion interferes with pyrimidine metabolism and DNA damage repair pathways. Preclinical data demonstrated that depletion of arginine by PEGylated arginine deiminase (ADI‐PEG 20) enhanced liposomal doxorubicin (PLD) cytotoxicity in cancer cells with argininosuccinate synthase 1 (ASS1) deficiency. The objective of this study was to assess safety and tolerability of ADI‐PEG 20 and PLD in patients with metastatic solid tumors.

**Methods:**

Patients with advanced ASS1‐deficient solid tumors were enrolled in this phase 1 trial of ADI‐PEG 20 and PLD following a 3 + 3 design. Eligible patients were given intravenous PLD biweekly and intramuscular (IM) ADI‐PEG 20 weekly. Toxicity and efficacy were evaluated according to the Common Terminology Criteria for Adverse Events (version 4.0) and Response Evaluation Criteria in Solid Tumors (version 1.1), respectively.

**Results:**

Of 15 enrolled patients, 9 had metastatic HER2‐negative breast carcinoma. We observed no dose‐limiting toxicities or treatment‐related deaths. One patient safely received 880 mg/m^2^ PLD in this study and 240 mg/m^2^ doxorubicin previously. Treatment led to stable disease in 9 patients and was associated with a median progression‐free survival time of 3.95 months in 15 patients. Throughout the duration of treatment, decreased arginine and increased citrulline levels in peripheral blood remained significant in a majority of patients. We detected no induction of anti‐ADI‐PEG 20 antibodies by week 8 in one third of patients.

**Conclusion:**

Concurrent IM injection of ADI‐PEG 20 at 36 mg/m^2^ weekly and intravenous infusion of PLD at 20 mg/m^2^ biweekly had an acceptable safety profile in patients with advanced ASS1‐deficient solid tumors. Further evaluation of this combination is under discussion.

## Graphical abstract

This combination therapy with ADI‐PEG 20 and Doxil had a reversible and manageable toxicity profile, and patients tolerated the treatment. Tumor shrinkage and prolonged duration of therapy were observed in several patients.

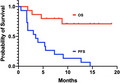

## 1 INTRODUCTION

One promising strategy for treating malignancies is amino acid deprivation based on the observation that tumors are auxotrophic for otherwise nonessential amino acids because of genetic defects.^1^ Arginine is a semiessential amino acid involved in the regulation of numerous cellular processes.^2^, ^3^, ^4^, ^5^ Most normal human cells can synthesize arginine from citrulline via two key enzymes: argininosuccinate synthase 1 (ASS1) and argininosuccinate lyase. Cancer cells that are deficient in the necessary enzymatic pathway for arginine synthesis must instead obtain arginine from the blood for growth and survival.^6^, ^7^

As a result of the observations regarding the potential anticancer activity of arginine depletion described above, researchers focused on the development of arginine deiminase (ADI) for treatment of auxotrophic cancer. Synthesis of ADI with polyethylene glycol of 20,000 molecular weight via a succinimidyl succinate linker (ADI‐PEG 20) proved to result in the optimal therapeutic effect via an enhanced half‐life and diminished immunogenicity in previous phase 1 and 2 studies.^8^, ^9^, ^10^ The clinical development of ADI‐PEG20 as a monotherapy was hampered after a randomized placebo controlled phase III clinical trial of second line ADI‐PEG20 as a single agent did not demonstrate an overall survival (OS) or progression‐free survival (PFS) benefit in patients with sorafenib‐refractory advanced hepatocellular carcinoma.^11^ Further preclinical studies^12^, ^13^ and clinical trials^14^, ^15^, ^16^ have focused on ADI‐PEG 20 in combination with other chemotherapeutic drugs.

Preliminary analysis of 149 breast tumor samples showed that 64% of the tumors were ASS1‐deficient. Across all major pathological subtypes of breast cancer, ASS1 expression served as an independent prognostic factor for poor OS. Furthermore, an in vitro cell proliferation study with MDA‐MB‐231 breast cancer cells showed a synergistic therapeutic effect of ADI‐PEG 20 and doxorubicin.^17^, ^18^ The liposomal form of doxorubicin (PLD) is associated with less cardiac toxicity than non‐formulated doxorubicin^19^ and is a preferred single agent for chemotherapy for recurrent or metastatic breast cancer. Therefore, we conducted the present phase 1 trial of ADI‐PEG 20 plus PLD in patients with HER2‐negative metastatic breast cancer or other advanced solid tumors harboring ASS1 deficiency.

## 2 PATIENTS AND METHODS

This single‐center, open‐label, phase 1 clinical trial of ADI‐PEG 20 in combination with PLD following a standard 3 + 3 design was approved by the Institutional Review Board at The University of Texas MD Anderson Cancer Center (NCT01948843). Written informed consent was obtained from each patient before study entry.

### 2.1 Patient selection

Patients aged 18 years or older with an Eastern Cooperative Oncology Group performance status of 0–2 and adequate organ function were eligible if they had HER2‐negative metastatic breast cancer or other advanced solid tumors harboring ASS1 deficiency detected using an immunohistochemical test conducted in a Clinical Laboratory Improvement Amendments‐certified molecular diagnostics laboratory at MD Anderson. Patients had progressive disease that was evaluable or measurable according to the Response Evaluation Criteria in Solid Tumors (version 1.1).^20^ For tumors previously treated with anthracyclines, time to treatment failure with an anthracycline‐containing regimen was at least 12 months. Patients were excluded if they had epirubicin exposure of greater than 600 mg/m^2^ or received a doxorubicin dose greater than 240 mg/m^2^, had significant or uncontrolled intercurrent illnesses, had history of another primary cancer (unless treated curatively or unlikely to affect outcome), had uncontrolled or progressing central nervous system metastases, had not recovered from prior cancer treatment, were allergic to PEGylated or *Escherichia coli* products, were pregnant or lactating, had a history of seizure disorder not related to the underlying cancer, received previous therapy with ADI‐PEG 20, had prior hypersensitivity to doxorubicin, and had serious infections requiring treatment with systemic antibiotics.

### 2.2 Treatment plan and assessment

This study followed a standard 3 + 3 design, with eligible patients receiving intramuscular (IM) ADI‐PEG 20 weekly and intravenous (IV) PLD biweekly. Intrapatient dose escalation was not allowed. One cycle of treatment was 4 weeks. Patients continued treatment until experiencing tumor progression or prohibitive toxic effects or withdrawing. After patients had received a cumulative doxorubicin and PLD dose greater than 500 mg/m^2^ or a combined cumulative epirubicin, doxorubicin, and PLD dose greater than 900 mg/m^2^, they were allowed to continue taking PLD at the discretion of the treating physician with frequent echocardiogram monitoring once every 6 weeks provided that they did not have a drop in left ventricular ejection fraction of at least 10%. Patients who could not receive PLD due to cumulative exposure were allowed to receive IM ADI‐PEG 20 with closely cardiac monitoring if they had no worse than stable disease. Toxicity and efficacy assessments were performed according to the Common Terminology Criteria for Adverse Events (version 4.0) and Response Evaluation Criteria in Solid Tumors (version 1.1), respectively.

### 2.3 ASS1 expression, pharmacodynamics, and immunogenicity

Baseline formalin‐fixed, paraffin‐embedded tumor or fresh core biopsy samples obtained from the patients were assessed for ASS1 expression via immunohistochemical staining. ASS1 deficiency was defined as immunohistochemical staining in less than 50% of tumor cells as described previously.^8^ Peripheral blood samples were obtained before ADI‐PEG 20 every 2 weeks (on even weeks) and 4 h after treatment every 2 weeks (on odd weeks) each treatment and assessed weekly for levels of arginine and citrulline and titers of anti‐ADI‐PEG 20 antibodies for 24 weeks. Circulating arginine and citrulline levels were determined using liquid chromatography‐mass spectrometry (LC‐MS), and plasma anti‐ADI‐PEG 20 antibody levels were assessed using an enzyme‐linked immunosorbent assay‐based immunogenicity assay.^9^

### 2.4 Statistical analysis

This study followed the 3 + 3 design, with an additional 3 patients allowed per dose level as needed for safety assessment. Descriptive summary statistics were used to characterize demographics, safety, and antitumor activity. Categorical data were summarized using frequencies and percentages. Continuous data were summarized by medians with 95% CIs and ranges. Progression‐free survival (PFS) and OS were estimated using the Kaplan–Meier method. PFS was defined as the time from the first day of the study treatment to death or progression. Patients without evidence of progression or death were censored at the date of last radiographic assessment of progression. OS was defined as the time from enrollment to death or December 19, 2016, at which time patient data were censored. Statistical inferences were based on two‐sided tests, and *p* values <0.05 were considered significant. Statistical analyses were carried out using SPSS software (version 22; IBM).

## 3 RESULTS

### 3.1 Patient demographics

We enrolled a total of 15 patients from May 2014 to August 2015. They had metastatic solid tumors consisting of HER2‐negative breast (*n* = 9), adenoid cystic (*n* = 4), non–small cell lung (*n* = 1), and mucoepidermoid (*n* = 1) carcinomas (Table 1). Patients received 18 mg/m^2^ IM ADI‐PEG 20 weekly and 15 mg/m^2^ IV PLD biweekly (*n* = 3; dose level 1), 18 mg/m^2^ IM ADI‐PEG 20 weekly and 20 mg/m^2^ IV PLD biweekly (*n* = 3; dose level 2), 36 mg/m^2^ IM ADI‐PEG 20 weekly and 15 mg/m^2^ IV PLD biweekly (*n* = 3; dose level 3), or 36 mg/m^2^ IM ADI‐PEG 20 weekly and 20 mg/m^2^ IV PLD biweekly (*n* = 6; dose level 4).

**TABLE 1:** Patient baseline characteristics (*n* = 15)

Characteristic	No. (%)
Median age (range)	51 years (31–59)
Gender	
Male	5 (33)
Female	10 (67)
Race	
White	11 (73)
African‐American	3 (20)
Asian	1 (7)
Pathology	
Breast cancer	9 (60)
Adenoid cystic carcinoma	4 (26)
Non–small cell lung cancer	1 (7)
Mucoepidermoid carcinoma	1 (7)
Eastern cooperative oncology group performance status	
0	3 (20)
1	12 (80)
Prior anthracycline	7 (47)
Prior systemic therapy	15 (100)
Median prior therapies (range)	4 (2–9)
Prior surgery	15 (100)
Prior radiation	12 (8)

### 3.2 Safety

We evaluated all patients for the safety of the study treatment. We observed no dose‐limiting toxicities or treatment‐related deaths. Treatment‐related adverse events (AEs) occurred in 14 patients (93%). Two patients (13%) experienced AEs leading to treatment discontinuation. Eight patients (53%) had treatment‐emergent grade 3/4 AEs. Six patients were lost to follow‐up. Nine patients (60%) died of malignant disease progression. None of the deaths were due to AEs, and none died within 30 days of discontinuation of the study treatment.

As shown in Table 2, the most commonly reported AEs were neutropenia (*n* = 10 [67%]), neutropenia (*n* = 9 [60%]), anemia (*n* = 4 [27%]), palmar‐plantar erythrodysesthesia syndrome (*n* = 5 [33%]), fatigue (*n* = 4 [27%]), urinary tract infection (*n* = 4 [27%]), and increased alanine aminotransferase level (*n* = 4 [27%]). Other frequently reported AEs included thrombocytopenia, nausea, stomatitis, pyrexia, and back pain. Of note is that three patients had safely received high cumulative doses of anthracyclines. One patient who had received 240 mg/m^2^ doxorubicin prior to study entry safely received 880 mg/m^2^ PLD in our study. Another patient with prior exposure to 450 mg/m^2^ epirubicin received 210 mg/m^2^ PLD. The third patient without prior exposure to anthracycline‐based regimens received 450 mg/m^2^ PLD. We observed no clinically meaningful left ventricular ejection fraction declines or electrocardiography changes in these 15 patients.

**TABLE 2:** Treatment‐related adverse event with ADI‐PEG20 and Doxil (*n* = 15)

Adverse event	Dose level 1 (*n* = 3)		Dose level 2 (*n* = 3)		Dose level 3 (*n* = 3)		Dose level 4 (*n* = 6)		Total (*n* = 15)	
	Any grade	≥Grade 3	Any grade	≥Grade 3	Any grade	≥Grade 3	Any grade	≥Grade 3	Any grade	≥Grade 3
Neutropenia	1 (33%)	0	3 (100%)	0	2 (67%)	2 (67%)	4 (67%)	3 (50%)	10 (67%)	5 (33%)
Anemia	1 (33%)	0	0	0	1 (33%)	0	2 (33%)	0	4 (27%)	0
Thrombocytopenia	0	0	0	0	1 (33%)	0	2 (33%)	0	3 (20%)	0
Alkaline phosphatase increased	1 (33%)	0	0	0	2 (67%)	0	1 (17%)	1 (17%)	4 (27%)	1 (7%)
Back pain	0	0	2 (67%)	0	0	0	1 (17%)	0	3 (20%)	0
Constipation	1 (33%)	0	1 (33%)	0	0	0	1 (17%)	0	3 (20%)	0
Cough	0	0	1 (33%)	0	1 (33%)	0	2 (33%)	0	4 (27%)	0
Fatigue	1 (33%)	0	1 (33%)	0	1 (33%)	0	1 (17%)	0	4 (27%)	0
Nausea	0	0	1 (33%)	0	1 (33%)	0	1 (17%)	0	3 (20%)	0
Palmar‐plantar erythrodysaesthesia syndrome	1 (33%)	0	1 (33%)	1 (33%)	0	0	3 (50%)	0	5 (33%)	1 (7%)
Pyrexia	2 (67%)	0	1 (33%)	0	0	0	0	0	3 (20%)	0
Stomatitis	0	0	2 (67%)	0	1 (33%)	0	0	0	3 (20%)	0
Urinary tract infection	1 (33%)	0	2 (67%)	0	0	0	1 (17%)	0	4 (27%)	0

Note:

Dose level 1 (ADI‐PEG 20 18 mg/m^2^ IM QW and Doxil 15 mg/m^2^ IV Q2W), 2 (ADI‐PEG 20 18 mg/m^2^ IM QW and Doxil 20 mg/m^2^ IV Q2W), 3 (ADI‐PEG 20 36 mg/m^2^ IM QW and Doxil 15 mg/m^2^ IV Q2W) and 4 (ADI‐PEG 20 36 mg/m^2^ IM QW and Doxil 20 mg/m^2^ IV Q2W).

### 3.3 Efficacy and survivals

**FIGURE 1:**
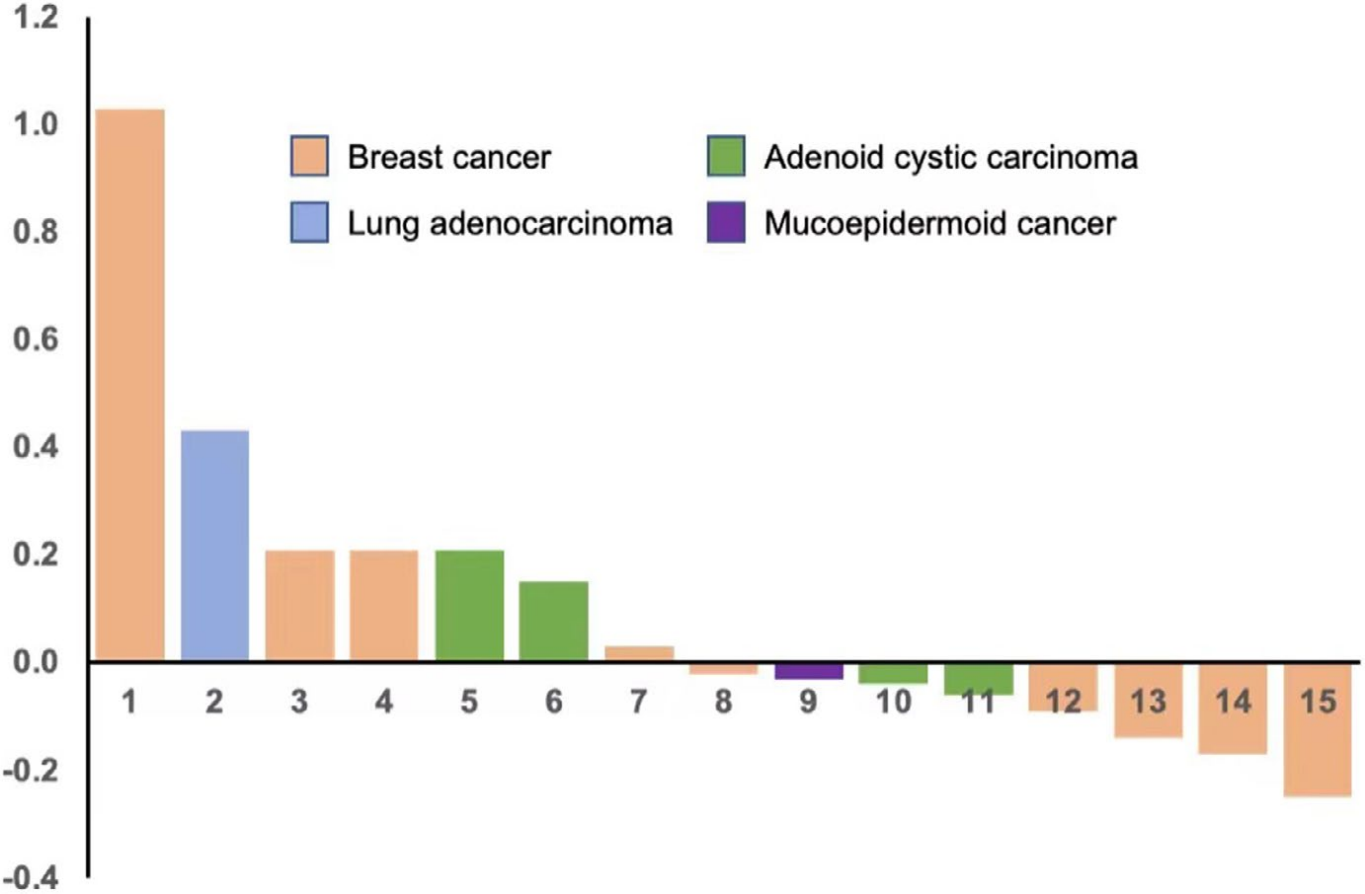
A waterfall plot shows best tumor responses in 15 patients with metastatic ASS1 deficient solid tumors treated with ADI‐PEG20 and liposomal doxorubicin

**FIGURE 2:**
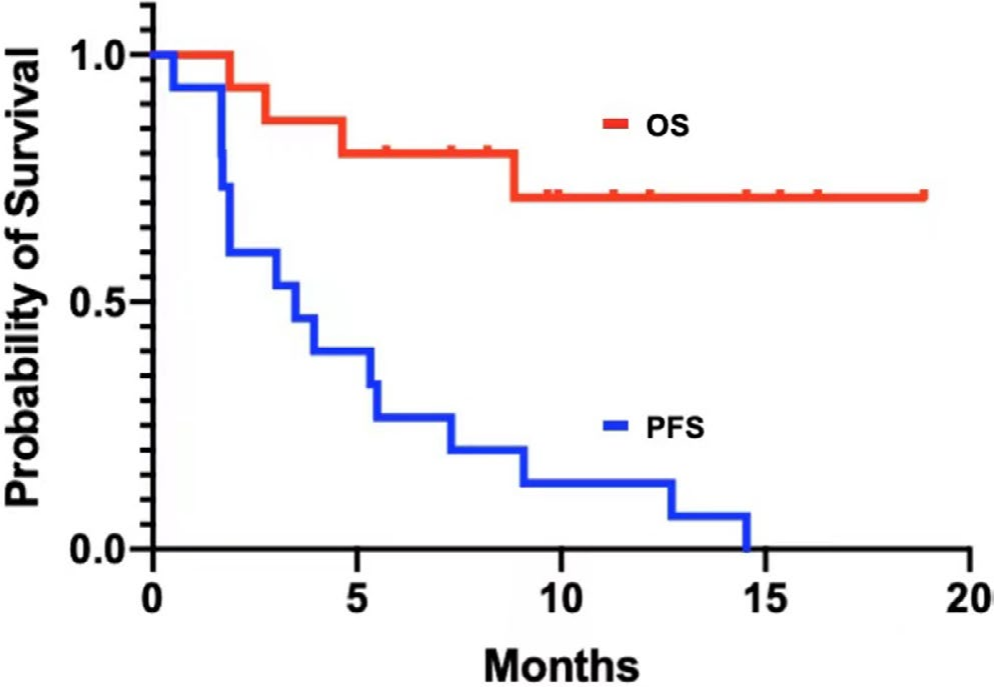
Kaplan–Meier curves of progression survival (PFS, blue, 3.95 months, 95% confidence interval, 0.86–7.04) and overall survival (OS, red, not evaluable with 11 patients censored) in 15 patients with metastatic ASS1 deficient solid tumors treated with ADI‐PEG20 and liposomal doxorubicin

In the 15 patients given ADI‐PEG 20 and PLD, we observed no objective responses but did observe tumor shrinkage (Figure 1), with 3 patients having greater than 10% tumor reduction and 9 patients who had stable disease. In this cohort of patients, the median PFS time was 3.95 months (95% CI, 0.86–7.04 months), and the 6‐month PFS rate was 33%. The median OS time was not evaluable since 11 patients were censored at the cutoff date of this study (Figure 2).

### 3.4 Pharmacodynamic and immunogenicity analyses

**FIGURE 3:**
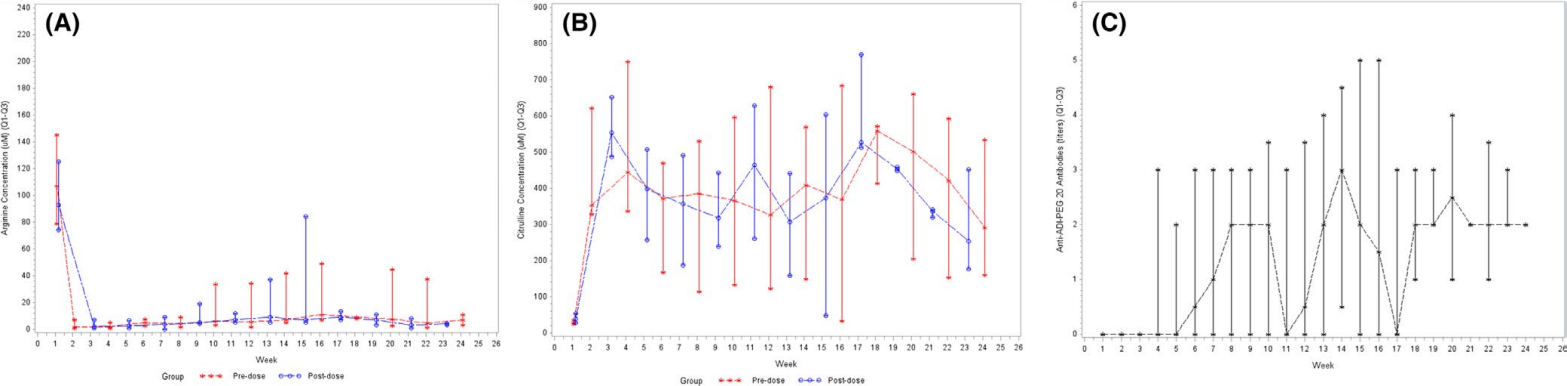
Pharmacodynamic changes detected in the plasma obtained from the study patients (*n* = 15): (A) Median arginine levels in peripheral blood by timepoint; (B) Median citrulline levels in peripheral blood by timepoint; (C) Anti‐ADI‐PEG20 antibody levels in peripheral blood by timepoint

Pharmacodynamic studies showed that treatment with ADI‐PEG 20 rapidly induced durable depletion of arginine from peripheral blood. As shown in Figure 3A, mean levels of arginine declined rapidly after administration of the first dose of the study treatment (118.5 μM at baseline to 3.3 μM prior to cycle 1 day 8). Arginine levels remained low at all time points analyzed. Accordingly, citrulline levels in peripheral blood increased rapidly after administration of the first dose of the study treatment (31.0 μM at baseline to 486.2 μM prior to cycle 1 day 8) and remained elevated as shown in Figure 3B. Induction of anti‐ADI‐PEG 20 antibodies occurred as early as week 4 of the study in 10 patients (Figure 3C). We could not determine whether antibody levels were correlated with arginine depletion. In five patients, we did not detect induction of antibodies throughout the treatment period.

## 4 DISCUSSION

We made several unique observations in this study. First, the combination therapy with IM ADI‐PEG 20 given at 36 mg/kg weekly and IV PLD given at 20 mg/m^2^ biweekly is safe. We observed no dose‐limiting toxicities in all 15 patients. Second, although we saw no objective responses, we observed tumor shrinkage in six patients given treatment for more than 24 weeks. Third, a patient with adenoid cystic carcinoma received the treatment for 14.9 months, and another with breast cancer received it for 20.5 months. Neither patient had clinically meaningful cardiac toxicity, with left ventricular ejection fraction changes of about 5%. Fourth, we confirmed that the treatment with ADI‐PEG 20 and PLD had optimal biological effects on arginine metabolism as demonstrated by rapidly and persistently decreasing levels of arginine and increasing levels of citrulline in peripheral blood.

ASS1 is expressed in a wide variety of normal cells. In patients with ASS1‐deficient malignancies, researchers have observed several adverse clinicopathological features, poor differentiation, increased tumor size, and lymph node involvement, associated with poor prognosis.^21^, ^22^ In the treatment of ASS1‐deficient cutaneous melanoma, uveal melanoma, and malignant pleural mesothelioma, ADI‐PEG 20 was well tolerated and demonstrated antitumor activity.^9^, ^10^, ^23^, ^24^ Based on preclinical data demonstrating that ADI‐PEG 20 combined with chemotherapy synthetically killed cancer cells harboring ASS1 deficiency, investigators have explored several combination therapeutic regimens in a number of clinical studies.^15^, ^25^, ^26^, ^27^ To the best of our knowledge, our study described herein is the first reported study of ADI‐PEG 20 in combination with liposomal doxorubicin for cancer therapy.

Our experience with ADI‐PEG 20 and PLD confirmed previous observations that PLD is associated with less cardiac toxicity than is nonformulated doxorubicin.^28^ The proportion of grade ≥3 AEs in our study was markedly worse than previously reported for PLD alone or with other cytotoxic agents.^29^, ^30^ However, all these AEs in the present study were generally reversible and manageable. Among the many potential reasons for this observation is that the patients in the present study were heavily pretreated, as 13 of the 15 patients had received 3 or more lines of therapy.

We did not observe antitumor activity of ADI‐PEG 20 and PLD similar to that in previous clinical studies of ADI‐PEG 20 combined with other chemotherapeutic agents, which induced effective doubling of the response rate in patients with ASS1‐deficient malignant tumors.^15^, ^16^, ^27^ We showed that our combination therapy led to stable disease in 60% of the patients and was associated with a median PFS time of 3.95 months, which was comparable with or better than those for other regimens as fourth‐line or later treatment of HER2‐negative breast cancer.^31^, ^32^, ^33^

The pharmacodynamic activity of ADI‐PEG 20 was evidenced by rapid and durable depletion of arginine from peripheral blood. Of note, arginine reduction and citrulline elevation induced by ADI‐PEG 20 combined with PLD were more prolonged than those observed previously with ADI‐PEG 20 alone or combined with other chemotherapeutic agents.^16^, ^23^, ^26^ We did not detect anti‐ADI‐PEG 20 antibodies throughout the treatment period in some patients. This may explain the prolonged biological activity of ADI‐PEG 20, but this should be evaluated further in larger studies.

In considering the clinical relevance of our findings, several limitations should be borne in mind. First, the small sample sizes limited the validity of statistical assessments of safety and efficacy. Secondly, patient selection bias may limit the generalizability of our findings, as it does for many clinical trials. Thirdly, OS was estimated with 11 patients being censored at the cut‐off date of this study. This estimated survival curve may misinterpret the real survivals in these 15 patients.

In conclusion, we observed no dose‐limiting toxicities in patients who received ADI‐PEG 20 and PLD. This combination therapy had a reversible and manageable toxicity profile, and patients tolerated the treatment. Tumor shrinkage and prolonged duration of therapy were observed in several patients. The dose level of IM ADI‐PEG 20 at 36 mg/m^2^ weekly and IV PLD at 20 mg/m^2^ biweekly may be considered the recommended phase 2 doses, which is supported by their optimal pharmacodynamic effects. Further evaluation of this combination in treatment of selective tumors or specific lines of therapy is under discussion.

## CONFLICTS OF INTEREST

Amanda Johnston and John Bomalaski are employees of Polaris Pharmaceuticals, Inc.

## AUTHORS CONTRIBUTIONS

Shuyang Yao: Literature search, data assembly, data interpretation, analysis, and manuscript writing. Filip Janku: Study design, data collection, data interpretation, and writing. Kimberly Koenig: Study design, data interpretation, analysis, and writing. Apostolia Maria Tsimberidou: Study design, data interpretation, analysis, and writing. Sarina Anne Piha‐Paul: Study design, data interpretation, analysis, and writing. Nai Shi: Study design, data collection, data assembly, data analysis, and writing. John Stewart: Data collection, data interpretation, and writing. Amanda Johnston: Study design, data interpretation, analysis, and financial support. John Bomalaski: Study design, data interpretation, analysis, and financial support. Funda Meric‐Bernstam: Study design, data interpretation, analysis, and writing. Siqing Fu: Literature search, study design, administrative support, data collection, data interpretation, analysis, and writing. Final approval of manuscript: All authors. Accountable for all aspects of the work: All authors.

## ETHICAL STATEMENT

No formal ethics approval was applicable in our study. This clinical study was reviewed and approved by the Institutional Review Board (IRB) at the University of Texas MD Anderson Cancer Center. The study was performed according to the principles defined by the Declaration of Helsinki and Good Clinical Practice guidelines. All patients gave written informed consent prior to any study‐related procedure.

## Data Availability

All data supporting the results in this manuscript are available at Polaris Pharmaceuticals, Inc.
